# Antepartum Mastitis: A Case Report

**DOI:** 10.1155/S1064744995000275

**Published:** 1995

**Authors:** Michelle Smith-Levitin, Daniel W. Skupski

**Affiliations:** Department of Obstetrics and Gynecology Division of Maternal-Fetal Medicine Cornell University Medical Center/New York Hospital 525 E. 68th Street, Room J 130, New York NY 10021 USA

## Abstract

*Background:* Acute mastitis commonly occurs in the postpartum period. It has been reported only rarely in the antepartum period.

*Case:* A 14-year-old patient presented at 29 weeks gestation with her symptoms and examination consistent with bilateral mastitis that had worsened over 2 months. She had evidence of systemic infection. She was treated with parenteral antibiotics and local skin care. She gradually improved and delivered a healthy infant at term.

*Conclusion:* The management of antepartum mastitis can be derived from experience with puerperal mastitis. It must include early recognition, a search for predisposing factors and causative organisms, and aggressive treatment. Such an approach can lead to successful pregnancy outcome with minimal fetal or maternal morbidity.


Infectious Diseases in Obstetrics and Gynecology 3:34–36 (1995)
© 1995 Wiley-Liss, Inc.

### Antepartum Mastitis: A Case Report

**Michelle Smith-Levitin and Daniel W. Skupski**

*Department of Obstetrics and Gynecology, Cornell University Medical Center-New York Hospital, New York, NY*

Abstract

***Background:*** **Acute mastitis commonly occurs in the postpartum period. It has been reported only rarely in the antepartum period.**

***Case:*** **A 14-year-old patient presented at 29 weeks gestation with her symptoms and examination consistent with bilateral mastitis that had worsened over 2 months. She had evidence of systemic infection. She was treated with parenteral antibiotics and local skin care. She gradually improved and delivered a healthy infant at term.**

***Conclusion:*** **The management of antepartum mastitis can be derived from experience with puerperal mastitis. It must include early recognition, a search for predisposing factors and causative organisms, and aggressive treatment. Such an approach can lead to successful pregnancy outcome with minimal fetal or maternal morbidity.** © 1995 Wiley-Liss, Inc.

Key words

Breast infection, pregnancy, parenteral antibiotics

Acute mastitis associated with pregnancy is a common problem with an estimated incidence of 1–9%.[1–4] The majority of the cases occur postpartum, usually within 6 weeks of delivery.[2,3,5] The occurrence of acute mastitis in the antenatal period is uncommon to rare. Only 3 cases have been reported in the obstetrical literature in the past 20 years, probably representing underreporting.[1,6] We report a case of chronic sporadic mastitis occurring during gestation that required hospitalization and IV antibiotics.

### CASE REPORT

A 14-year-old primigravida presented for prenatal care at 29 weeks gestation, complaining of bilateral breast pruritus for 2 months. She reported that her breasts had become progressively more red and sore. She had received no prenatal care or medical attention for her breast symptoms prior to this visit. She had no significant medical, surgical, or family history, and no known allergies. She had come to the United States from the Philippines 1 month earlier but denied any known infectious or environmental exposures, illicit drug use, breast trauma, or manipulation other than scratching. There was no historical or physical evidence of abuse.

Her temperature was 37.5°C (normal). Bilateral excoriations, ulcerations, and erythema were noted on the surface of the breasts. There were also bilateral induration involving the areola and breast tissue (9 × 7-cm area on the right and 3 × 2-cm area on the left), purulent drainage from both nipples, and bilateral axillary adenopathy. The uterine fundus was nontender and measured 26 cm. The remainder of the physical exam was within normal limits.

The initial laboratory studies showed a leukocyte count of 18,300 with 78% neutrophils and 11% lymphocytes. The hematocrit was 27% and the hemoglobin was 8.4 g/dl. The platelet count was

**Address correspondence/reprint requests to Dr. Michelle Smith-Levitin, Department of Obstetrics and Gynecology, Division of Maternal-Fetal Medicine, Cornell University Medical Center/New York Hospital, 525 E. 68th Street, Room J130, New York, NY 10021.**

Received January 3, 1995

Accepted March 31, 1995

483,000/μl. The chemistry and electrolyte panels were normal. Electronic fetal monitoring revealed a reactive nonstress test and no uterine contractions. Ultrasound showed a single, active, intrauterine pregnancy consistent with 29 weeks gestation. A diagnosis of chronic antepartum bilateral mastitis was made. The patient was admitted to the obstetrical service for parenteral antibiotic therapy with nafcillin (2 g q 4 h), metronidazole (500 mg q 6 h), and aggressive local care with hot soaks and twice daily wet-to-dry dressing changes.

Subsequently, hepatitis studies, syphilis serology, glucose challenge test, and cervical cultures for gonococcus and chlamydia returned negative. A culture from the nipple discharge grew *Staphylococcus aureus* that was resistant to penicillin but sensitive to most other antibiotics including nafcillin. The patient remained afebrile throughout her hospitalization, and her leukocytosis resolved. Her breast examinations slowly showed improvement. On the 7th hospital day, the skin ulcerations were granulating, the areas of induration were markedly decreased in size and tenderness, and there was only scant serous drainage from the right breast. The patient was discharged on oral dicloxacillin (500 mg q 6h) for 3 weeks and continued local breast care.

The remainder of the patient's antepartum course was unremarkable. She had a spontaneous vaginal delivery at 41 weeks gestation of a live female infant weighing 3,440 g with Apgar scores of 8 and 9. At the time of delivery, she still had lesions on her breasts but they were crusted over without evidence of active infection (no induration, erythema, or fluctuance). She elected not to breast-feed.

**TABLE 1. Possible causes and predisposing factors in antepartum mastitis**

| |
|---|
| IV-drug abuse |
| "Skin popping" via dilated mammary veins |
| Physical abuse |
| Rough sexual contact |
| Cigarette or other burns |
| Human bites |
| Cultural rituals |
| "Toughening" the nipples |
| Abrasions from "coining" |
| Application of caustic substances (alcohol, benzoin) |
| Dermatologic conditions (pruritus) |
| Fungal infections |
| Eczema |
| Pruritic urticarial papules and plaques of pregnancy |
| Cholestasis of pregnancy |
| Granulomatous disease |
| Tuberculosis |
| Actinomycosis |
| Sarcoidosis |
| Inflammatory breast carcinoma |
| Milk (or colostrum) stasis |
| Spontaneous (idiopathic) |
| Supernumerary or axillary breast tissue |

### DISCUSSION

Infectious mastitis occurring in the puerperium is most often a consequence of milk stasis or cracking of the areolar skin which allows bacteria to gain entry and cause mammary adenitis or cellulitis and potentially abscess formation.[2] Such a consistent predisposing factor is less clear in the few reported cases of antepartum mastitis.[1,6] Table 1 lists some of the possible causes and predisposing factors. It is important that these be investigated so that the patient can be educated toward prevention. A thorough history, including social service evaluation, and physical examination of our patient made substance abuse, physical abuse, and cultural rituals unlikely. We believe that she had a dermatitis, possibly secondary to a superficial candidal infection that developed into a cellulitis as a result of the entry of bacteria through excoriations from her scratching. The untreated cellulitis eventually progressed to a full-blown mastitis. To our knowldege, this is the first report of a case of mastitis associated with pregnancy that presented with an indolent, chronic course.

Early treatment of mastitis has clearly been shown to decrease the incidence of abscess formation.[3,5] Traditionally, puerperal mastitis has been treated with oral antibiotics along with pumping or continued nursing to facilitate milk drainage.[2,3] Pumping cannot be done in cases of antepartum mastitis because of the potential for initiating preterm labor. Prevention of abscess formation and bacteremia is indicated in the gravid patient as these conditions may also lead to premature labor and delivery. These serious sequelae may be avoided with the use of parenteral antibiotics.[1]

The initial choice of antibiotics is empirical. A failure of the patient to respond and culture results can be used to modify the regimen. *S. aureus*, which is often β-lactamase positive, is the most commonly cultured organism in studies of puerperal mastitis.[2,5,7] A penicillinase-resistant antibiotic such as nafcillin, which is acceptable for use in pregnancy (class B)[8] and is well tolerated, is a good choice. Niebyl and colleagues[5] reported the successful treatment of mastitis with penicillin even when the organism recovered was resistant. The reason for this unexpected response is not known. We prefer a penicillinase-resistant antibiotic as it also provides coverage for some of the less commonly involved organisms such as *S. epidermis*, α-hemolytic streptococci, group A β-hemolytic streptococci, and nonhemolytic streptococci.

Rare organisms such as *Salmonella bredeney*[6] and gram-negative organisms (*Klebsiella pneumoniae*[5] and *Serratia marcescens*[1]) have been reported to cause antepartum mastitis. Thus, a Gram's stain and culture of the nipple discharge prior to initiating antibiotic therapy are musts. In a patient whose history makes an obscure organism more likely, as with a human bite,[1] consideration should be given to broadening the coverage to include anaerobic and gram-negative bacteria.

When an abscess has been diagnosed, surgical drainage and debridement are necessary.[6] An abscess can be difficult to ascertain amidst the extensive inflammation seen with mastitis alone. This was demonstrated by our patient in whom large areas of induration could have been mistaken clinically for an abscess. Thus, metronidazole was included in the initial therapeutic regimen. Hot compresses can expedite resolution of the induration, making more clear the diagnosis of mastitis without abscess.

If a patient does not respond rapidly to treatment, a more aggressive search for an abscess or an underlying mass is indicated. Needle aspiration is useful in diagnosing granulomatous mastitis[9] and in obtaining material for culture in a patient whose nipple discharge is scant or lacking. Tissue for histology should also be obtained in refractory cases to exclude inflammatory carcinoma.

The recognition that mastitis can occur antenatally and a targeted investigation to elicit predisposing factors are the keys to diagnosis. The management must be early and aggressive to prevent complications. Our report illustrates that such an approach can result in successful treatment with minimal maternal or fetal morbidity.

### REFERENCES

1. Wong MK, Smith CV, Phelan JP: Antepartum mastitis: A report of two cases. J Repord Med 31:511–513, 1986.
2. Marshall BR, Hepper JK, Zirbel CC: Sporadic puerperal mastitis: An infection that need not interrupt lactation. JAMA 233:1377–1379, 1975.
3. Devereux WP: Acute puerperal mastitis: Evaluation of its management. Am J Obstet Gynecol 108:78–81, 1970.
4. Filton AA: Incidence of puerperal and lactational mastitis in an industrial town of some 43,000 inhabitants. Br Med J 19:693–696, 1945.
5. Niebyl JR, Spence MR, Parmley MD: Sporadic (nonepidemic) puerperal mastitis. J Reprod Med 20:97–100, 1978.
6. Stamm AM: *Salmonella bredeney* mastitis during pregnancy. Obstet Gynecol 59:29S–30S, 1982.
7. Matheson I, Aursnes I, Horgen M, Aabo O, Melby K: Bacteriological findings and clinical symptoms in relation to clinical outcome in puerperal mastitis. Acta Obstet Gynecol Scand 67:723–726, 1988.
8. Briggs GG, Freeman RK, Yaffe SJ: Drugs in Pregnancy and Lactation. 3rd ed. Baltimore: Williams & Wilkins, p 440, 1990.
9. Macansh S, Greenberg M, Barraclough B, Pacey F: Fine needle aspiration cytology of granulomatous mastitis: Report of a case and review of the literature. Acta Cytol 34:38–42, 1990.

## References

[PDF_00227] Devereux W. P. (1970). Acute puerperal mastitis. Evaluation of its management.. Am J Obstet Gynecol.

[PDF_00244] Macansh S., Greenberg M., Barraclough B., Pacey F. (1990). Fine needle aspiration cytology of granulomatous mastitis. Report of a case and review of the literature.. Acta Cytol.

[PDF_00224] Marshall B. R., Hepper J. K., Zirbel C. C. (1975). Sporadic puerperal mastitis. An infection that need not interrupt lactation.. JAMA.

[PDF_00237] Matheson I., Aursnes I., Horgen M., Aabø O., Melby K. (1988). Bacteriological findings and clinical symptoms in relation to clinical outcome in puerperal mastitis.. Acta Obstet Gynecol Scand.

[PDF_00232] Niebyl J. R., Spence M. R., Parmley T. H. (1978). Sporadic (nonepidemic) puerperal mastitis.. J Reprod Med.

[PDF_00235] Stamm A. M. (1982). Salmonella bredeney mastitis during pregnancy.. Obstet Gynecol.

[PDF_00222] Wong M. K., Smith C. V., Phelan J. P. (1986). Antepartum mastitis. A report of two cases.. J Reprod Med.

